# Machine Learning Classification of Axillary Lymph Nodes Using Microwave Signals

**DOI:** 10.3390/s26144466

**Published:** 2026-07-14

**Authors:** Daniela M. Godinho, João M. Felício, Carlos A. Fernandes, Raquel C. Conceição

**Affiliations:** 1 Instituto de Biofísica e Engenharia Biomédica, Faculdade de Ciências, Universidade de Lisboa, Campo Grande, 1749-016 Lisbon, Portugal; rcconceicao@ciencias.ulisboa.pt; 2 Instituto de Telecomunicações, Instituto Superior Técnico, Universidade de Lisboa, 1049-001 Lisbon, Portugal; joao.felicio@lx.it.pt (J.M.F.); carlos.fernandes@lx.it.pt (C.A.F.)

**Keywords:** Axillary Lymph Nodes, breast cancer, classification, machine learning, microwave imaging

## Abstract

Axillary Lymph Nodes (ALNs) can be affected by breast cancer, and the number of affected ALNs is a determinant factor in breast cancer staging. Microwave imaging (MWI) has emerged as a promising technique for ALN assessment, addressing limitations in conventional imaging modalities. This study investigates, for the first time, the classification of ALNs and axillary regions from microwave signals, without image reconstruction. Classification is performed considering realistic morphological characteristics of ALNs reported in the literature and is based solely on geometric differences, which differ from targets previously explored in microwave-based classification studies. Eighty ALN numerical models were mathematically generated based on state-of-the-art anatomical descriptions. Microwave signals were simulated for three scenarios of different complexity, involving one and two ALNs, representing healthy and metastasised conditions. The methodology evaluated multiple combinations of signal types, feature extraction methods, and classifiers, including scenarios with multiple targets, reflecting clinically relevant axillary conditions and limited angular views inherent to axillary imaging. Classification accuracy reached 95% for single-ALN scenarios using kNN, while more complex two-ALN cases achieved accuracies up to 83.3% using SVM. These results demonstrate the potential of microwave signal-based classification to differentiate healthy and metastasised ALNs and axillary regions, supporting future integration with MWI image interpretation.

## 1. Introduction

Breast cancer accounted for more than 2.3 million new cases in 2022 [1] and, in 2023, represented 31% of cancer cases and 15% cancer deaths in women in the United States [2]. Axillary Lymph Nodes (ALNs) drain most of the lymph from the breast and, in more advanced stages of cancer, can drain cancer cells from breast tumours. In those cases, the survival rate of breast cancer patients can drop from around 95% to 10% [3]. The number of metastasised ALNs is evaluated for breast cancer staging, thus impacting treatment decisions. However, when assessing ALNs, current imaging modalities present limitations in performance [4], showing a variable range of sensitivity from 55% to 97%, and specificity from 49% to 86% [5,6]. To date, the Sentinel Lymph Node Biopsy (SLNB) is considered the most accurate technique to diagnose ALNs, which is an invasive and costly procedure [7]. However, studies show that in approximately 70% of the cases, SLNB could have been avoided since it yields a negative result [8,9].

Microwave Imaging (MWI) has shown promising results for early detection of breast cancer and brain strokes [10,11] and has recently been studied to aid the diagnosis of ALNs [12,13]. MWI consists of illuminating the region of the body of interest with an electromagnetic pulse and recording the backscattered signals using one or multiple antennas. Strong backscatters result from the presence of dielectric differences between tissues at microwave frequencies. Several studies have measured the dielectric properties of ALNs [13,14,15]. Although an MRI-based study has estimated a dielectric contrast of 31% between healthy and metastasised nodes [16], it is not clear whether that contrast, together with the low resolution of MWI, is enough to differentiate them in reconstructed microwave images.

Classification algorithms have been widely used by the MWI community to assist the interpretation of microwave images and signals. As the main information in microwave images is the reflectivity map of the region of interest, classification methods can offer complementary objective information, which may potentially contribute to more accurate ALN diagnosis. This non-invasive method is crucial in clinical settings. Classification algorithms have been used to assess detected targets in MWI, e.g., to distinguish between healthy breasts and those with tumours [17,18,19,20] and between benign and malignant breast tumours [21,22,23,24,25], to distinguish between Intracerebral Haemorrhage (ICH) from Ischaemic Strokes (IS) [26,27,28], and to detect brain tumours [29]. More recently, studies to monitor Alzheimer’s disease with electromagnetic data have emerged [30,31], relying solely on classification results rather than also using imaging results.

Distinct shapes and sizes characterise benign and malignant breast tumours, with benign tumours typically exhibiting round shapes and malignant tumours presenting spiculated shapes. Microwave signals are affected by these morphological features, and differences between the two tumour types were identified [32,33]. Conversely, ICH and IS are only distinguishable by their dielectric properties, where IS often presents a lower dielectric constant than healthy tissues and ICHs have a higher dielectric constant than healthy tissues. The possibility of using microwave-scattered signals to detect differences between healthy and metastasised ALNs, e.g., in terms of shape, has never been reported in the literature.

This paper presents a numerical study in which different classification strategies were evaluated to assess how size and shape differences of ALNs reported in the literature influence the microwave signals. Healthy ALNs are characterised by a kidney-shaped appearance, with their dimensions varying between 1 and 25mm in their longest axis [34]. Metastasised ALNs are usually larger and more spherical. Studies show that the ratio between the longest (L) and shortest (S) axes can be used to classify ALNs: e.g., L/S<1.7 and S>9mm typically occur for metastasised ALNs [35]. The absence of the hilum structure, located in the convexity of the ALN, is another characteristic of metastasised ALNs [35]. Considering these characteristics, 80 models of ALNs were created to simulate microwave signals in numerical scenarios of varying complexity. Then, a classification pipeline, with different methods and two objectives, was applied: (1) distinguish healthy and metastasised ALNs; (2) distinguish healthy axillary regions and axillary regions with metastasised ALNs.

Feasibility studies of classification of tumour presence using microwave signals started with simplified scenarios in simulation [17] and experimental environments [18]. Increasing levels of complexity have been tested more recently, such as considering anatomically more realistic breast models and heterogeneity variability [19,20]. Recently, classification approaches have been used in breast MWI prototypes under clinical trials and patient studies [36,37,38]. Differences between studies have been mostly related to realistic modelling and testing scenarios, while authors have shown that state-of-the-art classification strategies remain suitable for medical microwave signal classification. Some studies have considered the classification of cases with multiple tumours [39], but none of these studies have considered multiple tumours of different types. For brain MWI classification, strokes have been modelled as spheres, and methodologies have ranged from extracting features from segmentation of microwave images [26] and using a graph approach to retrieve features from microwave signals [27]. One or two brain tumours of the same type (with similar characteristics) were considered in some studies [29,40]. However, the approach in these studies was significantly different from previous ones, as actual microwave images were used to train deep learning algorithms.

The literature has shown the potential of classification algorithms to aid MWI interpretation [41,42], presenting methodologies which can also be applied for ALN classification, using microwave signals acquired with MWI prototypes, based on their morphological features. However, most studies consider only a single target, a scenario that is unlikely in the axillary region, which contains multiple ALNs that can be either healthy or metastasised. Also, the characteristics that differentiate healthy from metastasised ALNs are distinct from the considered targets (breast tumours or brain lesions). In all these studies for breast and brain MWI, the microwave scatter can be recorded 360° around the target. However, the specific geometry of the axillary region limits the angular view to a maximum of 90°, which consequently reduces the number of backscattered signals for imaging of ALNs. Therefore, evaluating the feasibility of ALN classification becomes imperative to complement an MWI-based diagnosis. To the best of the authors’ knowledge, no prior research on lymph node classification using direct microwave signals without the need to reconstruct the image has ever been performed in the context of ALNs. The first steps towards this objective were performed in [43] and further extended in this publication.

This study aims to demonstrate the feasibility of classifiers and Machine Learning (ML) algorithms in axillary MWI scenarios. This will help to define future directions of research for axillary MWI and, if feasible, increase the potential of axillary MWI in clinical practice.

In summary, the novel contributions of this paper include the following:

- The first study on the classification of ALNs using microwave signals considers their morphological characteristics reported in the literature, which are different from targets considered in previous classification studies using microwave signals.
- The first classification study used microwave signals acquired in a body part with a limited angular view when compared to breast or brain MWI applications and, therefore, limited information.
- Classification of scenarios with multiple targets using microwave signals, considering different numbers of healthy and diseased targets, emphasising the importance of also considering multiple targets.
- Performance comparison of several combinations of types of signals, feature extraction methods and classifiers.

In Section 2, the methodology used to create the ALN numerical models is presented, as well as the classification pipeline, including the feature extraction methods and classification algorithms. In Section 3, the classification results of the different simulated scenarios are presented. Section 4 provides an overall discussion, outlining the limitations of the study and potential directions for future work. And, finally, in Section 5, the final conclusions are drawn and a summary of suggestions for future work is presented.

## 2. Material and Methods

### 2.1. Numerical Models and Setup

Numerical simulations were considered in this feasibility study, since ensuring correct 3D printing of ALN models with variable shapes and sizes can be challenging. This section explains how the ALN models were created and presents the classification scenarios of healthy and metastasised ALNs that were considered and simulated, and the corresponding simulation parameters.

#### 2.1.1. Axillary Lymph Nodes Modelling

A dedicated mathematical formulation was used to create the ALN models used in this study, enabling the creation of multiple models with diverse shapes and sizes. Extracting such variability from medical exams, such as MRI exams, would be a time-consuming process and would require a large dataset of both healthy and metastasised ALNs. Although some efforts have been performed to create MRI-based ALN numerical models [16] (which are available in an open-access repository), they do not present sufficient variability. Several studies have described the sizes and shapes of ALNs [35,44,45,46]; however, to the best of the authors’ knowledge, no numerical modelling for ALNs has ever been proposed.

**Figure 1:**
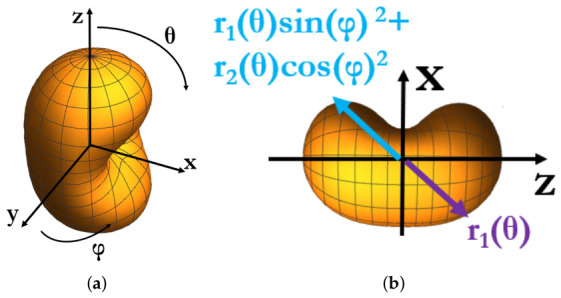
Illustration of the spherical coordinates considered in the creation of the numerical ALN models in (**a**) 3D view and (**b**) in the xz-plane.

Firstly, 28 numerical models of healthy and metastasised ALNs of different shapes were created using Wolfram Mathematica^®^, version 12.0 by implementing the following equations:

(1) $$r_{1}\left(\theta \right)=1-a\cdot {\left[\sin\theta \right]}^{b}$$

(2) $$r_{2}\left(\theta \right)=1-c\cdot \exp\left(-{\left(\frac{\theta -\theta_{0}}{d}\right)}^{2}\right)$$

(3) $$r\left(\theta ,\phi \right)=\left\{\begin{array}{cc} r_{1}\left(\theta \right), & \mathrm{if}\frac{\pi }{2}<\phi <\frac{3\pi }{2}\\ r_{1}\left(\theta \right){\left[\sin\phi \right]}^{2}+r_{2}\left(\theta \right){\left[\cos\phi \right]}^{2}, & \mathrm{otherwise} \end{array}\right.$$

where the convexity of the model, mainly observed in the negative *x*-axis (Figure 1), is defined by parameters *a* and *b*. The ALN hilum is approximately represented by a concavity. The depth, shape and location of the concavity observed in the positive *x*-axis can be changed by the parameters *c*, *d* and $\theta_{0}$ changing, respectively. To obtain a proper ALN model, suitable combinations of the 5 parameters need to be found. This was performed by trial and error, to match the actual shapes of ALNs [35], while considering the following parametric bounds: *a* between 0 and 2π/3; *b* and *c* between 0 and 1; *d* between 0 and 3; and $\theta_{0}$ between 0 and 2π. After the definition of parameters, each model was exported as an STL file and imported to the simulation environment without any surface modification. The defined parameters for the 28 models are presented in Supplementary Material S1.

**Figure 2:**
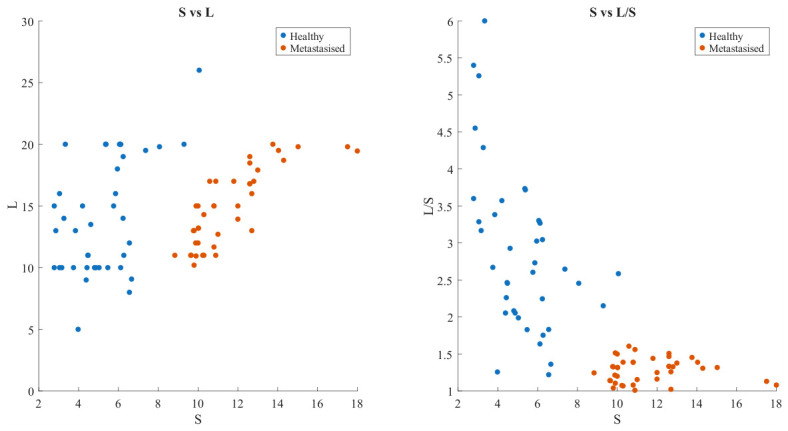
Scatter plots showcasing the relationship between the longest axis (L), shortest axis (S), and L/S ratio of all ALN models.

**Figure 3:**
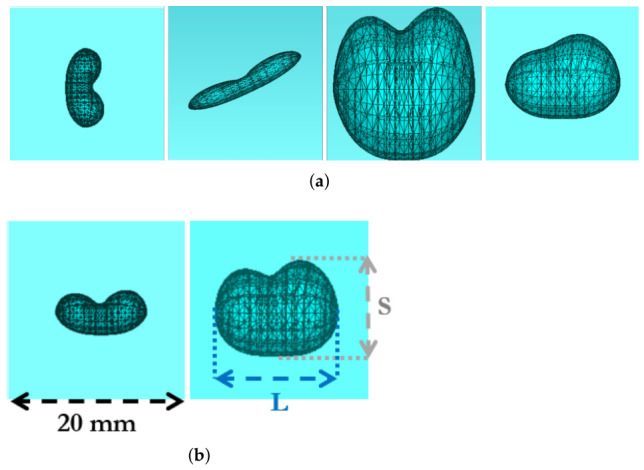
(**a**) Examples of healthy (two on the left) and metastasised (two on the right) ALN models, within a $20\times 20\times 20\;{\mathrm{mm}}^{3}$ cube, and (**b**) a representation of how the longest axis (L) and shortest axis (S) were measured.

A total of 80 ALN models, equally distributed between healthy and metastasised cases, were derived from the 28 parent models through resizing (differently in each axis) and rotation operations. The morphological characteristics defining healthy and metastasised ALNs were respected following the previously reported criteria in Section 1: healthy ALNs present L/S≥1.7 or S<9mm; a concavity, representing the hilum, was included in healthy ALN models, a feature that may not be present in metastasised ALNs. It is stressed, though, that in a clinical scenario, this trait is not necessarily observed in all ALNs. The basic statistics of all models contained in the full dataset are shown in Table 1, and the distribution of their dimensions is shown in Figure 2. Figure 3 shows examples of generated healthy and metastasised ALN models and how ALN dimensions were measured. The dimensions of each resulting model, including the performed transformations applied to the parent models, and agreement with the morphological characteristics are presented in Supplementary Material S2.

#### 2.1.2. Simulation Scenarios

**Figure 4:**
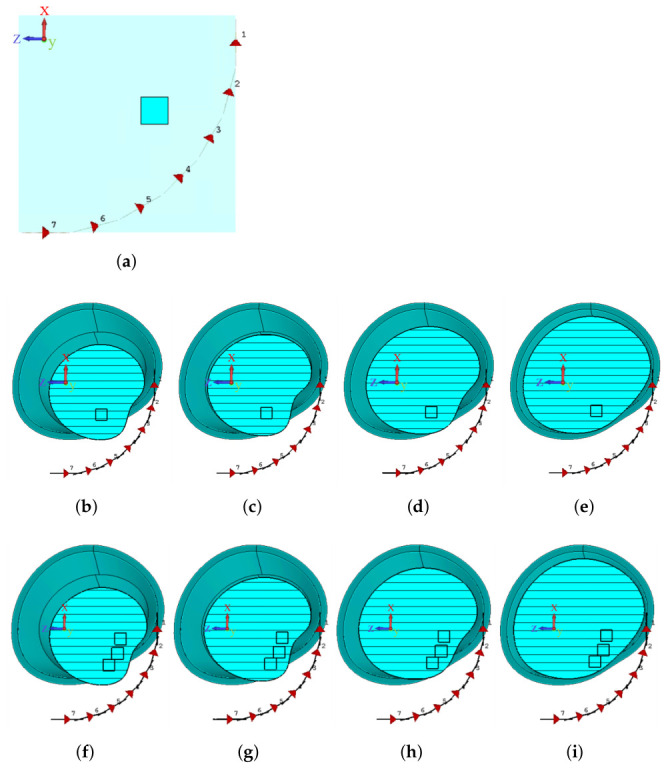
Representations of (**a**) the simulated scenario where the antenna and the ALN were embedded in the same medium (scenario A), (**b**–**e**) four different xz-planes of the axillary region phantom containing a single ALN (scenario B), and (**f**–**i**) four different xz-planes of the axillary region phantom containing multiple ALNs (scenario C). The planes in (**b**–**i**) are spaced 20 mm apart along the *y*-axis. The red arrows represent the feed point of each antenna position, while the small blue square represents the $20\times 20\times 20\;{\mathrm{mm}}^{3}$ volume where each ALN was placed. Numbers 1–7 refer to the antenna positions within each plane.

A monostatic simulated system was used to evaluate three scenarios with progressively increasing complexity. As the axillary region is part of the torso and ALNs are superficial on one of the sides of the torso, the antenna’s positioning is limited by an angular view of 90°, as shown in the setups presented in Figure 4.

The signals were calibrated in all scenarios by subtracting the signals obtained by simulating the antenna and the phantoms and the signals obtained by simulating only the antennas, i.e., free-space [47].

In Scenario A, the classification performance of each ALN was evaluated without other confounders. To this end, the antenna and the ALN were embedded in a dielectric medium that mimicked the properties of adipose tissue (Figure 4a). The antenna was scanned at 7 evenly-spaced positions along a quarter of a circumference with a radius of 140mm within the same plane of the ALN. This radius was established based on the observations in [12], which allows a feasible scan around an irregular axillary region. Also, to minimise confounders affecting the backscattered signals, the ALN model was always centred in a $20\times 20\times 20\;{\mathrm{mm}}^{3}$ cube in order to control their positioning and the numerical mesh. The average distance between the ALN centres and the antenna positions was 74.5mm, varying between 46 and 120mm. As the ALN model is in a fixed position between simulations, this varying distance between the ALN centres and antenna positions should not influence the classifier performance. A total of 80 simulations were performed, where one of the 80 ALNs was considered for simulation at a time.

Scenario B consisted of a dielectric phantom with adipose tissue-mimicking properties and a complex shape resembling a realistic axillary region, including concave and convex features. The CST modelling tools were used to create this shape, using as reference the MRI-based model presented in [13]. MRI-based shaped phantoms, and consequently more complex shaped phantoms, were not used for this study, as it would substantially increase the computational time and complexity of the simulations. The dielectric phantom had a frustum-of-a-cone shape with a concavity on one side. It included two parallel bases, representing cross-sections at the arm/elbow and part of the torso. The radius varies along its height, ranging from 120mm to 260mm, with a total vertical height of 150mm. The antenna was scanned in air along a quarter-circular trajectory with a radius of 155mm around the phantom. To increase the variability of the air-phantom interface and the relative position of ALNs with respect to the surface, the antenna and ALN locations were assigned to four different planes of the axillary region phantom each time (Figure 4b–e). This approach was intended to better represent the anatomical variability across different axillary region phantoms. The distance between the ALN centres and the nearest phantom surface varied between 29 and 46mm, which is within the range of ALN depth reported in the literature (14 to 80mm) [48], while the distance between the phantom surface and the antenna positions ranged from 13 to 82mm. Scenario B comprised 7 angular antenna positions in each of the 4 planes, i.e., 28 antenna positions per ALN, and 80 simulated ALNs.

Scenario C considered the same pseudo-realistic axillary region phantom used in scenario B with multiple ALNs placed inside. This scenario was designed to evaluate the ability of the classifiers in distinguishing signals acquired in axillary regions containing only healthy ALNs from those containing metastasised ALNs. The antenna was also swept in 7 positions around the axillary region across four different planes, as shown in Figure 4f–i. Two ALNs were considered for each simulation, and they were placed in two out of three pre-defined positions. The positions for placing the ALNs were chosen randomly, ensuring that all possible combinations of ALN positions were chosen an equal number of times. The spacing between the ALNs depends on the specific pair of pre-defined locations where ALNs are placed, on the orientation of ALNs within each location and on the dimensions of each ALN. The distance between the ALN centres in adjacent positions was 25mm, indicating the gap between the ALNs’ surfaces can range from 0 to 50mm. Each simulation could include, specifically, 0, 1 or 2 metastasised ALNs. Each ALN model was used in one simulation only. This means the distribution of 80 ALN models was as follows: 13 simulations included 2 healthy ALNs; 14 simulations included 1 healthy and 1 metastasised ALNs; and 13 simulations included 2 metastasised ALNs. This allows adopting distinct classification tasks.

#### 2.1.3. Antennas

A planar slot-based single-layer printed antenna was used. The antenna consists of two crossed exponential slots and is referred as the XETS antenna [47]. This type of antenna exhibits a highly stable radiation pattern and phase centre, together with pure linear polarisation across its entire bandwidth [49]. A full near-field characterisation of this antenna is described in [47].

**Figure 5:**
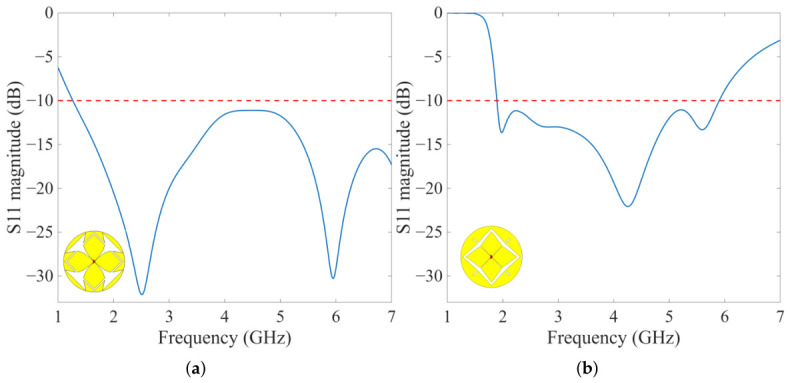
Schematic representation of the XETS antenna (in yellow) and the corresponding S-parameter magnitude response (dB) while operating (**a**) in adipose tissue and (**b**) in air. The red dashed line represents the −10dB threshold.

Several variations in this antenna have been presented in the literature [47]. In this work, two different designs were considered for the XETS, since in scenario A the antenna was immersed in a dielectric, while in scenarios B and C the antenna operated in air. They were both impedance matched (below −10dB) from 2 to 6GHz. The antenna designed to operate in adipose tissue-mimicking medium has a 17mm radius and 0.25mm thickness, while the antenna operating in air has a 28mm radius and 0.26mm thickness [47]. Figure 5 shows the configuration and corresponding input reflection curves of both antenna variants.

#### 2.1.4. Simulation Parameters

The numerical simulations were created and performed using Computer Simulation Technology (CST) Studio Suite^®^ 2019 software [50]. The Time-Domain Solver was used with a hexahedral mesh. Scenarios A, B and C were simulated in linearly spaced 1001 points within the frequency range from 1 to 8GHz. A Perfectly Matched Layer (PML) was considered as a boundary condition, with an estimated reflection level of $10^{-4}$, and considering an automatic minimum distance to structure of ≈λ/4 at a central frequency of 4.5GHz.

The dielectric properties of adipose tissue were modelled using a relative permittivity of $\epsilon_{r}=8$ and a dissipation factor of tan(δ)=0.1. Although previous studies suggested a dielectric contrast of 31% between healthy and metastasised ALNs [16], the worst-case scenario was considered where there is no contrast between both types of ALNs. Hence, the classification will rely solely on differences in ALN morphology, namely their shape and size. The models were assigned a relative permittivity of $\epsilon_{r}=40$ and a dissipation factor tan(δ)=0.1, and restricted by a $20\times 20\times 20\;{\mathrm{mm}}^{3}$ cube.

The standard parameters for mesh cells per wavelength were used: 25 cells near the model and 10 cells away from the model. The total number of cells varied depending on the used phantom, and the cells’ size varied between materials with different dielectric properties. Consequently, the simulation time varied between scenarios A, B, and C, and depended on the computer usage at the time of simulations. In scenario A, each simulation contained around 38 million cells, with the smallest and largest cells with approximately 0.07mm (≈λ/1071) and 1.15mm (≈λ/65), respectively. In scenario B, each simulation contained around 123 million cells, with the smallest and largest cells with approximately 0.24mm (≈λ/312) and 3.70mm (≈λ/20), respectively. In scenario C, each simulation contained around 155 million cells, with the smallest and largest cells with approximately 0.24mm (≈λ/312) and 3.73mm (≈λ/20), respectively. Scenarios A, B and C were simulated in 5, 46 and 35 days, respectively, in a machine with 2 Intel^®^ Xeon Gold 6136 CPUs @ 3 GHz, 2 NVIDIA^®^ Quadro^®^ GP100 and 256 GB of RAM memory. These simulation times show that it would not be feasible to simulate more complex and realistic scenarios.

### 2.2. Classification Pipeline

The classification workflow was implemented in MATLAB^®^ 2018b using the Statistics and Machine Learning Toolbox. Considering the variability of methods used in the literature, the objective was to evaluate different methods and find the optimal approach for this MWI application. Thus, a wide range of combinations of signal types, feature extraction methods (FEMs) and classifiers were evaluated. Simulated signals were first transformed into feature sets using an FEM, which were subsequently used for model training and evaluation. In scenarios A and B, the task was to distinguish between healthy and metastasised ALNs. In contrast, scenario C was used to create three binary models of three tasks: distinguishing entirely healthy axillary regions from those containing (1) at least 1 metastasised ALN (H-M); (2) exactly 1 metastasised ALN (H-M1); and (3) exactly 2 metastasised ALNs (H-M2).

Four different types of signals were considered before applying a FEM: reflection coefficients in both Time Domain (TD) and Frequency Domain (FD)—absolute, real and imaginary parts. The signals were restricted to the frequency range 2 to 6GHz, as it is the operating band of the XETS antenna. Each resulting signal had 572 samples. Each type of signal was compared in the remaining steps of the processing. Three FEMs were considered to extract the features from the signals: (1) RAW signals, i.e., each frequency or time sample was used as a feature; (2) the F25 customised features reported in [24,51]; (3) Principal Component Analysis (PCA), where 1 to 20 components were used as features. The F25 customised features in [24,51] showed promising results with the classification of multistatic MWI signals, but were not evaluated with a monostatic system. While RAW can be used as FEM [38] and does not require additional processing, PCA is commonly used [18,25,26,27] as it decreases computational costs while maintaining high performances. Although it is known that real and imaginary parts of the signals may have different information, a comparison of types of signals has never been attempted. Also considering TD signals and absolute FD signals complements the analysis.

Several classifiers, with different fundamentals, have been extensively used in existing literature for the purpose of classifying microwave signals [18,19,24,25]. Support Vector Machines (SVMs) are widely used, but they are computationally costly. As mentioned in [25], other algorithms may yield similar or better results. Because classifiers rely on different learning mechanisms, their effectiveness may vary according to the type of data being analysed. Therefore, seven classifiers were used to classify the microwave signals recorded with ALNs in an axillary region: Linear Discriminant Analysis (LDA), Quadratic Discriminant Analysis (QDA), k-Nearest Neighbours (kNN), Naïve Bayes (NB), Decision Trees (DT), Random Forests (RFO) and SVM. All these classifiers have hyperparameters that were optimised. Thus, a grid-search methodology was applied by testing a wide range of values that ensured a good compromise between the coverage of the values and a moderate computational time during the training process. The hyperparameters used in the grid search are shown in Appendix A.

The dataset was divided into training and testing sets in an approximate 3:1 ratio, ensuring a sufficient number of signals for training and testing. The training set included ALNs created from 20 of the 28 parent ALN models, while the testing set included ALNs created from the remaining 8 parent models. A Leave-One-Out (LOO) cross-validation (CV) was applied to the training set to find the best-performing model. Depending on the considered scenarios, the training set has different dimensions. LOO CV allows for a more reliable comparison of results between the different scenarios and, despite increasing the computational time when compared to K-fold CV, allows for robust validation when only a small amount of data is available. When adopting PCA as FEM, PCA is employed for each subset of the training set, and the resulting transformation is subsequently applied to the corresponding validation set, thereby preventing data contamination. The final performance of the model was assessed using the testing set.

The data was divided into groups, where each group comprised the monostatic signals recorded per single ALN (scenarios A and B) or from illuminating an axillary region with a combination of two ALNs (scenario C). This ensured all signals of one simulation were only present in one of the sets at each time. The training set had $N_{a}\times \left(N_{gtv}-1\right)$ signals, the validation set had $N_{a}$ signals and the testing set had $N_{a}\times N_{gt}$, where $N_{a}$ is the number of antennas per simulation, $N_{gtv}$ is the number of groups for training/validation and $N_{gt}$ is the number of groups for testing. Table 2 summarises the number of observations and groups used for each scenario.

The resulting performance metrics were calculated considering the predictions of all validation sets. Then, the results were analysed by examining the predictions of the signals recorded at each antenna position, treated as separate and independent observations (“independent signals”), and the majority vote of predictions from all antenna positions for each ALN (“grouped signals”). Balanced accuracy, F1-score and sensitivity were analysed as performance metrics. For balanced datasets, the balanced accuracy is equal to the standard accuracy metric. The 95% Confidence Intervals (CI) for each metric were calculated using the bootstrap method with 10,000 iterations. For conciseness, they are presented for the chosen models only. For each type of signal, the best-performing models were chosen based on the balanced accuracy of the “independent signals” between all classifiers (after optimising the hyperparameters) and FEMs. Statistical differences between classifiers were evaluated using a paired group-level bootstrap procedure. Then, the results of “grouped signals” were analysed case by case. The models with the highest accuracy with “grouped signals” were then used for evaluation with the testing set and using the three metrics, plus specificity.

By using this approach, one can find the optimal combination of types of signals, classifiers and FEMs that allows a more accurate ALN diagnosis. Ultimately, this optimal model could be trained and used to test newly acquired signals, providing objective information on the axillary region under examination.

## 3. Results

In this section, the classification results are presented for each scenario described in Section 2.1.2.

### 3.1. Training and Validation of Scenarios A and B

**Figure 6:**
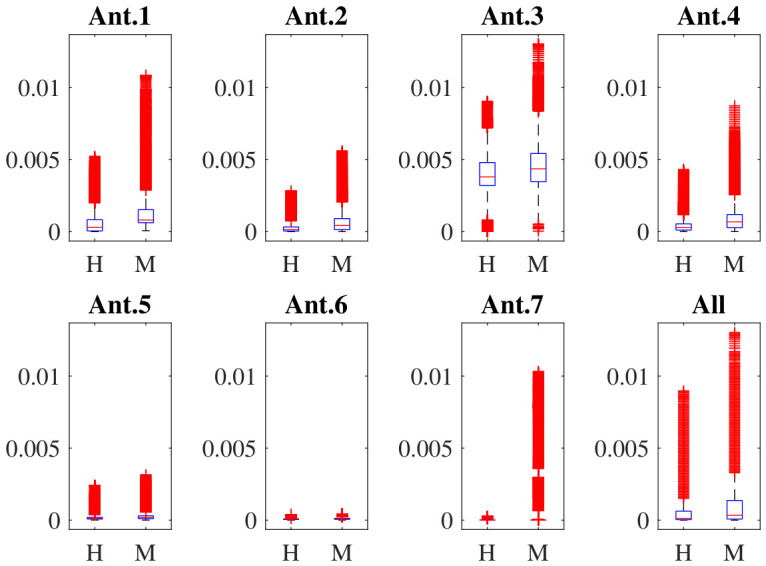
Box plots illustrating the distribution of the absolute FD signals for each class (H: healthy and M: metastasised) in scenario A and across the different antenna positions. The red line indicates the median, the boxes represent the first and third quartiles of the distribution. Red crosses denote outliers, defined as values located more than 1.5 times the interquartile range below the first quartile or above the third quartile.

Figure 6 shows the distribution of the magnitude of the absolute FD signals of scenario A over each class (healthy and metastasised). The magnitude values correspond to the values before applying any FEM. The box plots allow comparing the variability of magnitude values of the response of the two types of ALNs (healthy and metastasised) but only as an overall comparison of the samples (in this case, frequency samples). The magnitude values of 17,160 samples were used to create each box plot for each antenna position. This number of samples results from the 572 signal frequency samples of the 30 ALNs of each group (healthy and metastasised). For each box plot of all antenna positions, a total of 7×17,160 samples were considered. In general, visually, some differences are observed regarding the variability of the magnitude values between microwave signals recorded with healthy and metastasised ALNs. These box plots were created using all frequency points for visualisation. For statistical inference an aggregated ALN-level analysis was performed to preserve independence. The Mann–Whitney U test and rank-biserial r effect size test were applied to the mean magnitude values across frequency samples for each ALN. Results showed both healthy and metastasised groups are statistically different, with *p*-values ranging from $2\times 10^{-8}$ to $2\times 10^{-5}$, and rank-biserial r ranging between 0.64 and 0.84 for the different antennas.

**Figure 7:**
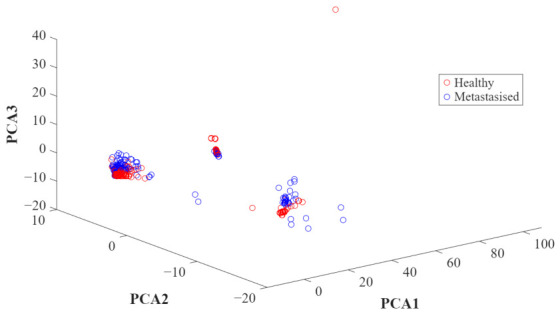
First 3 PCA components of absolute FD signals over each class (healthy and metastasised ALNs).

To better highlight how classifiers can differentiate both healthy and metastasised ALN groups, the first three PCA components of each class are shown in Figure 7. There is no clear separation between classes, but multiple sub-clusters can be observed within the data distribution. When a larger number of PCA components is considered, these sub-clusters can become more distinguishable, leading to improved classification performance.

Two types of results are presented—“independent signals” and “grouped signals”—as explained in Section 2.2. Table 3 shows the best performance results of “independent signals” of scenarios A and B after optimising the hyperparameters for each classifier. kNN is the classifier yielding the best results, followed by RFO and SVM in both scenarios A and B. The differences are not statistically significant between those. The accuracy results of LDA, QDA and NB (results only shown in the Appendix B.1) vary up to 10% for different types of signals and FEMs. In the majority of the cases, PCA outperforms F25 and RAW. A number of PCA components ranging from 16 to 20 yields the best results. In other cases, RAW may outperform PCA, but this improvement implies a high computational cost of the training process due to the considerably larger number of features. Among the best-performing models, the model with the highest accuracy also yielded the highest F1-score and sensitivity. When grouping the signals per ALN, the best models yielded an accuracy of 98.3% (95% CI = [94.4–100]%) (using kNN and independently of the type of signals) in both scenarios. In this case, only one ALN was misclassified as a false positive. The dimensions of this ALN, specifically L=20mm, which was the largest, and L/S=2.5, the lowest within those with the largest L.

### 3.2. Training and Validation of Scenario C

For conciseness, only the results with PCA are presented for scenario C. The performance results for kNN, RFO and SVM, after optimising the hyperparameters in the training/validation sets, are shown in Table 4, and for the remaining classifiers are shown in Table A4. The performance differences between kNN, RFO and SVM are not statistically significant. Unlike the previous scenarios, different types of signals yielded different accuracy results. When more than one ALN was included in the axillary region, the results indicate that the classification is more challenging. When compared to scenario B, accuracy values decrease almost 20%.

For the H-M model, RFO or SVM yielded the best performance results with the training/validation sets, outperforming kNN. These models were created with an unbalanced dataset where the chance level is 66.6%. When considering the independent signals, the SVM model with TD signals yielded the highest balanced accuracy, F1-score and sensitivity results, achieving 79.7% (95% CI = [76.8,82.7]%), 88.5% (95% CI = [86.5,90.3]%) and 92.3% (95% CI = [90.0,94.5]%), respectively. However, when grouping the signals, the classification performance improved when using the TD signals and kNN: a balanced accuracy, F1-score and sensitivity of 95.0% (95% CI = [83.3,100]%), 97.6% (95% CI = [91.4,100]%) and 100% (95% CI = [100,100]%), respectively. This means one axillary region was misclassified, namely, one false positive. The presence of false positives may indicate the model has the tendency for classifying the signals as the most prevalent class in the training dataset. The misclassified case did not present discernible patterns regarding the spatial location or size of the ALNs under evaluation.

The H-M1 models presented the lowest accuracy values for independent signals, but, unlike in H-M models, this is a balanced dataset with a chance level of 50%, so balanced accuracy is equal to accuracy. The model with the highest accuracy, using SVM and TD signals, resulted in a balanced accuracy, F1-score and sensitivity of 75.5% (95% CI = [72.0,79.0]%), 75.7% (95% CI = [71.5,79.4]%) and 76.1% (95% CI = [71.0,81.0]%) for independent signals, respectively. The accuracy when grouping the signals ranged from 75.0% to 90.0%, depending on the model. For the best model, a balanced accuracy, F1-score, and sensitivity of 90.0% (95% CI = [75.0,100]%), 90.9% (95% CI = [75,100]%), and 100% (95% CI = [100,100]%), respectively, were obtained, resulting in two false positive axillary regions. The predominant false positive among the models with the highest accuracy was one case where one ALN was larger than the other and the ALNs were positioned 25mm apart. The predominant common false negative consisted of one case where the healthy ALN had a longer longest axis when compared to the longest axis of the metastasised ALN, and the ALNs were also placed 25mm apart.

As anticipated, the H-M2 models, where the axillary regions exclusively contained one type of ALNs, yielded the highest accuracy results. Specifically, kNN and SVM yielded the most favourable performance metrics. The accuracy ranged from 85.0% to 100% when grouping the signals, achieving 100% when classifying the absolute part of FD signals and the TD signals. When there were misclassifications, up to two axillary regions were misclassified, either as false positives or false negatives depending on the model. No pattern was identified among the cases that were classified incorrectly. The highest performance model (using TD signals and SVM) yielded a balanced accuracy, F1-score and sensitivity of 81.1% (95% CI = [77.9,84.1]%), 78.8% (95% CI = [74.6,82.6]%) and 70.4% (95% CI = [64.9,75.6]%) for independent signals and correctly classified all models achieving 100% (95% CI = [100,100]%) for all metrics when grouping the signals.

### 3.3. Testing and Global Analysis

Table 5 shows the performance results of the best models when grouping the signals and using the testing set. The combination of types of signals, FEM and classifiers and their hyperparameters are also shown. The accuracy values are slightly worse than the ones obtained with the validation set especially in scenario C. These were obtained for a smaller dataset, which can explain the larger CIs in general. One must note the ALNs considered for the simulations of the testing set were created exclusively for this set, and the hyperparameters were not optimised considering these, which avoids data leakage. In all cases, specificity is more affected than sensitivity.

The results for scenario C, specifically for case H-M1, are not satisfactory. Specificity is always 0, and remaining metrics are very low, with very large CIs. However, this was the most difficult classification task, comparing healthy axillary regions with those with only ALN, which were not always in the same position. Also, as a limiting factor, 7 axillary regions were tested. The selected models of scenario C have misclassified cases in common. The three axillary regions misclassified in H-M (one false positive and two false negatives) were also misclassified in H-M1. In H-M1 two more false positives were observed. In H-M2, the only false positive was also misclassified in both H-M and H-M1. This specific case was an axillary region with a healthy ALN with a large longest axis L=20mm, but its L/S was lower than 1.7. No other patterns are observed in the remaining misclassified cases.

## 4. Discussion

The differences of the magnitude distribution of signals of scenario A (shown in Figure 6) are not substantial. Nevertheless, the responses recorded at each antenna position vary in terms of maximum magnitude and the dispersion of among magnitude values. These variations are primarily attributed to differences in the distances between the antenna position and the ALNs. This variability represents a real-case scenario where axillary regions present different shapes and ALNs are in different locations within the axillary region [52].

When considering one single ALN classification, the performance results vary among the four signal types. However, the differences are not substantial. The best accuracy values are comparable between both scenarios A and B. The same ALN was misclassified in scenarios A and B: the ALN with the largest L (L=20mm) and the lowest L/S within those with the largest L, which may explain the classifiers’ inability to correctly classify it. These findings suggest that the discriminative power of the microwave signals and, consequently, the classification performance, are not substantially affected by the complexity of scenario B. Nevertheless, these performance outcomes could be influenced by the inclusion of signals from the same axillary region plane in both training and testing sets. This implies information related to reflections at the air-dielectric interface may have been present in the training set and, consequently, the variability of the reflections did not affect the overall classification. As an alternative, classifiers could be used to train signals acquired in three planes and then be validated using data from the remaining plane. Using this approach (data shown in Table A5 of Appendix B.2), the results showed that the accuracy of “independent signals” decreases, but the accuracy of “grouped signals” remains almost unchanged for the best classifier, which means the majority of signals per ALN are still well classified. This reflects the challenges associated with examining an irregular shape such as the axillary region. However, in a clinical scenario, the “grouped signals” would be used, as only a single classification per case is required. Additionally, the variability of the considered ALN models may be limited, as the dataset was generated through transformations of only 28 parent ALNs created using the mathematical formulation presented in Section 2.1.1. When cross-validation is conducted, ensuring that all ALNs derived from the same parent model are placed in the same subset (training or validation), the accuracy decreases (Table A6 in Appendix B.2). Nevertheless, the performance obtained with grouped signals remains comparable, suggesting that the impact on the final classification results is minimal.

In both scenarios A and B, kNN was sufficient to obtain a good performance. These results are comparable to the tests reported in [25], with a breast MWI system, where kNN provided promising results. In contrast, in the present study, the number of PCA components was also optimised, while in [25] a fixed number of PCA components obtained with the Kaiser–Guttman test was used. For scenario C, where more information is present in the signals, more complex classifiers, such as SVM, are needed to achieve better performance. The findings also indicate that considering more than one ALN in the axillary region introduces multiple factors that can affect the classification of axillary regions as healthy and metastasised.

Table 6 shows a comparison of our study with state-of-the-art classification approaches to microwave data. It includes a variety of different tasks, types of data and protocols. Although metrics are not directly comparable, an overview of types of algorithms and achieved performances under different conditions is provided. In breast MWI, classifiers such as LDA, kNN, and SVM have been used to classify the presence of tumours and tumour type, using mostly PCA as a feature extraction method. Accuracy values up to 85% for tumour detection have been obtained for increasing levels of complexity in experimental scenarios [19,20]. Tumour classification (benign versus malignant) with experimental results [25] maintained similar accuracy levels (up to 92%) to simulation studies [22]. Tumour classification with clinical MWI data, surprisingly, achieved 88.5% accuracy considering features extracted directly from the microwave images [36] and 78% accuracy considering the real part of the recorded microwave signals of 113 patients as features [38]. Our study is the first one applied to the axillary region while evaluating different anatomical characteristics and presenting a limited field of view for the antennas (resulting in a reduced number of collected signals). Among the presented studies using simulation data, our study is the only one considering the real representation and behaviour of the antenna in the electromagnetic simulator, representing a more realistic scenario. This implies a higher computational cost and, thus, a more limited number of cases were evaluated.

The main limitations of our study are related to the modelling of ALNs and the axillary region. Specifically, a limited number of lymph nodes were considered (80 augmented models), and although scenarios of increased anatomical complexity were simulated, only up to two ALNs were simulated per axillary region, which does not fully reflect clinical reality. Additionally, ALN shapes were not derived from MRI data or other patient exams but instead based on descriptions reported in the literature [35]. Despite the reported differences in size and shape between healthy and metastasised ALNs—which was the assumption of our study—in practice these characteristics may overlap despite different pathology results [35]. Overlapping may arise either due to alterations in the hilum or the presence of micro-metastases, which remain challenging to identify using current imaging techniques. The limited number of ALN models has an impact on the performance of the classifiers tested in this study. These simplifications are consistent with the feasibility-oriented scope of this study, which aimed to investigate the classification of ALNs. Further evaluation under more realistic conditions is required in future studies.

The reliability of ML models in a real clinical setting relies on the representativeness of the training data. Thus, to translate this approach to real-world applications, our methodology would need to be applied to more complex scenarios, experimental data and, ultimately, patient data. When the complexity of the scenarios increases, by increasing the number of ALNs or inserting other anatomical structures such as skin or muscle, the classification performance is expected to be more affected. In particular, the dielectric response of surrounding tissues may mask ALNs’ response. The number of ALNs per axillary region can reach approximately 30 [53], which means it is unlikely that all of them can be individually detected by microwave-based systems. However, since level I ALNs are typically the first to be metastasised by breast cancer and are the shallowest [54], they are more likely to be within the detectable range of such systems.

In this context, the proposed approach in this paper is essential to find the proper type of signals and classification strategies that allow extracting more relevant information from the ALNs. The tests should be conducted step-by-step so the effects on classification can be explained. One should note that the considered methodology in this study was based on the worst-case scenario where there is no dielectric contrast between healthy and metastasised ALNs. Although there are indications that a dielectric contrast exists [16], further investigation is needed to quantify such contrast. Nevertheless, in a clinical scenario, it is expected that the existence of dielectric contrast improves the classification performance.

The quality of the implemented methodologies was ensured by following the best practices of ML to avoid over-fitting, using proper metrics to evaluate our models when using unbalanced datasets, and analysing the misclassifications and relating them to probable clinical observations. ML approaches focusing on microwave images instead of signals, such as those proposed by [26,29,36], could also be explored. However, false positive and skin artefact removal performance in imaging results would possibly greatly affect the performance of these ML models due to the limited angular view. Thus, the ultimate goal is to use the classification information to support clinicians in interpreting imaging results. Accordingly, further evaluation of the integration between classification and imaging outcomes should be performed. In a clinical context, an imaging result would be supplemented with the probability estimate of metastasised ALNs within the examined axillary region.

## 5. Conclusions

A feasibility study of classification to distinguish healthy from metastasised ALNs was performed using microwave simulated signals. For the first time, this study evaluated how classification algorithms using microwave signals perform under a limited angular view and with the particular shapes of ALNs.

Eighty ALN models were evaluated across increasingly complex scenarios using different signal types, FEMs and classifiers. Accuracy reached 95% when considering single ALNs, but accuracy dropped to 83.3% when considering multiple ALNs inside an axillary region. This suggests classifying ALNs and axillary regions is more challenging than classifying breast tumours due to the presence of multiple ALNs within a heterogeneous medium. Overall, these results indicate classifiers were able to differentiate healthy from metastasised ALNs, showing comparable performance to breast tumour classification and highlighting the potential of microwave data for axillary assessment. This type of approach provides more objective information to MWI results, supporting interpretation for clinicians and increasing the potential of axillary MWI’s adoption in clinical practice to improve breast cancer staging.

In future work, the performance of classifiers should be evaluated considering more complex anthropomorphic phantoms of the axillary region, considering skin and muscle tissues and multiple ALNs. Applying this methodology to experimental signals, considering real clinical conditions, is also important to improve the robustness of the methodology and models. Additionally, a large dataset of signals (hundreds of different cases) would be crucial to ensure the robustness of an ML model with increased sensitivity compared to standard imaging modalities. The methodology proposed in this study could be scaled to these more complex scenarios; thus, this paper strongly points towards valuable future directions. Ultimately, imaging results and classification performance should be jointly assessed to identify the situations in which they complement one another.

## Figures and Tables

**Table 1:** Dimensions of ALN models used in this study.

	Healthy ALNs (no. of Models = 40)		Metastasised ALNs (no. of Models = 40)	
	Mean	Range	Mean	Range
Longest-Axis (L) (mm)	14.1	5.0 to 26.0	14.8	10.0 to 20.0
Shortest-Axis (S) (mm)	5.1	3.0 to 10.0	11.8	10.0 to 18.0
L/S (mm)	2.9	1.1 to 6.7	1.3	1.0 to 1.6

**Table 2:** Distribution of signals per training/validation and testing sets for each scenario.

Scenarios		Training/Validation	Testing
A		420 signals (59 + 1 groups)	140 signals (20 groups)
B		1680 signals (59 + 1 groups)	560 signals (20 groups)
C	H-M	840 signals (29 + 1 groups)	280 signals (10 groups)
	H-M1	560 signals (19 + 1 groups)	196 signals (7 groups)
	H-M2	560 signals (19 + 1 groups)	168 signals (6 groups)

**Table 3:** Classification performance, measured by balanced accuracy (BAcc), F1-score (F1-S) and sensitivity (Sens) (%), for independent signals in scenarios A and B, after classifier hyperparameter optimisation with the training/validation sets. The PCA rows correspond to the optimal results obtained using 1 to 20 principal components. The best metrics for each type of signals are highlighted in bold.

Type of Signals	FEM	Scenario A			Scenario B		
		kNN	RFO	SVM	kNN	RFO	SVM
Absolute FD	RAW	**BAcc: 95.7** **F1-S: 95.9** **Sens: 99.0**	BAcc: 92.4 F1-S: 92.6 Sens: 94.8	BAcc: 84.5 F1-S: 85.8 Sens: 93.8	BAcc: 95.5 F1-S: 95.5 Sens: 97.1	BAcc: 91.5 F1-S: 91.7 Sens: 93.9	BAcc: **91.0** F1-S: 90.7 Sens: 88.1
	F25	BAcc: 90.7 F1-S: 91.1 Sens: 95.2	BAcc: 88.3 F1-S: 88.7 Sens: 91.9	BAcc: 82.4 F1-S: 82.0 Sens: 80.5	BAcc: 88.5 F1-S: 88.7 Sens: 89.8	BAcc: 87.9 F1-S: 88.3 Sens: 91.7	BAcc: 81.0 F1-S: 81.2 Sens: 81.8
	PCA	BAcc: 95.5 F1-S: 95.6 Sens: 98.1	BAcc: 91.7 F1-S: 92.1 Sens: 96.7	BAcc: 86.2 F1-S: 86.6 Sens: 89.0	**BAcc: 96.0** **F1-S: 96.0** **Sens: 97.6**	BAcc: 92.7 F1-S: 92.9 Sens: 96.0	BAcc: 91.6 F1-S: 92.0 Sens: 97.3
Real Part FD	RAW	BAcc: 94.5 F1-S: 94.7 Sens: 91.9	BAcc: 92.6 F1-S: 92.9 Sens: 96.2	BAcc: 86.7 F1-S: 86.3 Sens: 84.3	BAcc: 95.3 F1-S: 95.4 Sens: 97.5	BAcc: 91.1 F1-S: 91.3 Sens: 93.2	BAcc: 90.0 F1-S: 89.7 Sens: 87.5
	F25	BAcc: 92.4 F1-S: 92.8 Sens: 98.1	BAcc: 89.3 F1-S: 89.8 Sens: 94.3	BAcc: 84.0 F1-S: 83.8 Sens: 82.4	BAcc: 82.6 F1-S: 82.8 Sens: 83.4	BAcc: 88.7 F1-S: 88.9 Sens: 90.8	BAcc: 77.3 F1-S: 78.9 Sens: 84.9
	PCA	**BAcc: 95.2** **F1-S: 95.3** **Sens: 97.6**	BAcc: 91.4 F1-S: 91.8 Sens: 96.2	BAcc: 85.2 F1-S: 84.3 Sens: 79.5	**BAcc: 96.4** **F1-S: 96.5** **Sens: 98.2**	BAcc: 91.8 F1-S: 92.0 Sens: 94.8	BAcc: 92.5 F1-S: 92.7 Sens: 95.5
Imag. Part FD	RAW	**BAcc: 95.2** **F1-S: 95.4** **Sens: 98.6**	BAcc: 93.1 F1-S: 93.2 Sens: 95.2	BAcc: 85.2 F1-S: 84.9 Sens: 83.3	BAcc: 95.5 F1-S: 95.6 Sens: 97.5	BAcc: 92.3 F1-S: 92.5 Sens: 95.1	BAcc: 90.5 F1-S: 90.2 Sens: 87.6
	F25	BAcc: 94.0 F1-S: 94.2 Sens: 96.2	BAcc: 89.5 F1-S: 90.1 Sens: 95.2	BAcc: 83.8 F1-S: 81.9 Sens: 83.5	BAcc: 93.0 F1-S: 93.2 Sens: 95.2	BAcc: 89.3 F1-S: 89.6 Sens: 92.0	BAcc: 86.3 F1-S: 86.0 Sens: 83.8
	PCA	BAcc: 95.2 F1-S: 95.4 Sens: 98.1	BAcc: 92.9 F1-S: 93.2 Sens: 88.6	BAcc: 86.2 F1-S: 83.0 Sens: 81.4	**BAcc: 96.7** **F1-S: 96.8** **Sens: 98.5**	BAcc: 93.0 F1-S: 93.2 Sens: 95.7	BAcc: 95.0 F1-S: 94.9 Sens: 93.6
TD	RAW	BAcc: 92.9 F1-S: 93.1 Sens: 95.7	BAcc: 87.4 F1-S: 88.0 Sens: 92.4	BAcc: 84.3 F1-S: 84.4 Sens: 84.8	BAcc: 94.9 F1-S: 95.0 Sens: 96.7	BAcc: 87.5 F1-S: 88.0 Sens: 91.9	BAcc: 90.5 F1-S: 90.8 Sens: 93.7
	F25	BAcc: 92.1 F1-S: 92.2 Sens: 92.9	BAcc: 87.4 F1-S: 88.0 Sens: 92.4	BAcc: 84.3 F1-S: 84.0 Sens: 82.4	BAcc: 90.3 F1-S: 90.3 Sens: 90.0	BAcc: 86.8 F1-S: 87.2 Sens: 89.4	BAcc: 84.9 F1-S: 85.2 Sens: 87.1
	PCA	**BAcc: 94.5** **F1-S: 94.7** **Sens: 98.6**	BAcc: 92.1 F1-S: 92.5 Sens: 97.1	BAcc: 85.0 F1-S: 85.2 Sens: 83.3	**BAcc: 95.6** **F1-S: 95.6** **Sens: 97.1**	BAcc: 92.1 F1-S: 92.4 Sens: 95.7	BAcc: 91.9 F1-S: 92.1 Sens: 95.6

**Table 4:** Classification performance, measured by balanced accuracy (BAcc), F1-score (F1-S) and sensitivity (Sens) (%), for independent signals in scenario C, after classifier hyperparameter optimisation with the training/validation sets and when using PCA-X as FEM. The best metrics for each type of signals are highlighted in bold.

Comparison	Type of Signals	kNN	RFO	SVM
H-M	Absolute FD	BAcc: 74.1 F1-S: 79.9 Sens: 75.7	BAcc: 73.5 F1-S: 85.4 **Sens: 90.9**	**BAcc: 77.4** **F1-S: 86.9** Sens: 90.5
	Real Part FD	BAcc: 70.6 F1-S: 78.2 Sens: 75.2	BAcc: 71.7 **F1-S: 83.7** **Sens: 88.0**	**BAcc: 77.3** F1-S: 77.1 Sens: 66.4
	Imag. Part FD	BAcc: 70.8 F1-S: 78.2 Sens: 74.8	BAcc: 69.8 F1-S: 82.9 **Sens: 87.9**	**BAcc: 74.5** **F1-S: 84.5** Sens: 87.1
	TD	BAcc: 77.1 F1-S: 82.7 Sens: 79.5	BAcc: 75.7 F1-S: 86.7 Sens: 92.1	**BAcc: 79.7** **F1-S: 88.5** **Sens: 92.3**
H-M1	Absolute FD	BAcc: 69.8 F1-S: 64.9 Sens: 55.7	BAcc: 69.3 F1-S: 67.0 Sens: 62.5	**BAcc: 73.0** **F1-S: 74.5** **Sens: 78.6**
	Real Part FD	BAcc: 65.0 F1-S: 60.2 Sens: 52.9	BAcc: 68.0 F1-S: 65.9 Sens: 61.8	**BAcc: 70.2** **F1-S: 71.4** **Sens: 74.6**
	Imag. Part FD	BAcc: 65.0 F1-S: 60.6 Sens: 53.9	BAcc: 68.4 F1-S: 66.8 Sens: 63.6	**BAcc: 72.0** **F1-S: 73.2** **Sens: 76.8**
	TD	BAcc: 69.8 F1-S: 66.5 Sens: 60.0	BAcc: 70.9 F1-S: 69.3 Sens: 65.7	**BAcc: 75.5** **F1-S: 75.7** **Sens: 76.1**
H-M2	Absolute FD	**BAcc: 78.4** F1-S: 76.9 Sens: 71.8	BAcc: 77.0 F1-S: 76.2 Sens: 73.6	BAcc: 76.4 **F1-S: 78.4** **Sens: 85.7**
	Real Part FD	BAcc: 74.8 F1-S: 73.6 Sens: 70.4	BAcc: 72.7 F1-S: 72.2 Sens: 71.1	**BAcc: 75.5** **F1-S: 76.7** **Sens: 80.7**
	Imag. Part FD	BAcc: 75.9 F1-S: 74.6 Sens: 70.7	BAcc: 75.5 F1-S: 74.6 Sens: 71.8	**BAcc: 76.8** **F1-S: 76.7** **Sens: 76.4**
	TD	BAcc: 78.2 F1-S: 77.2 Sens: 73.6	BAcc: 76.1 F1-S: 75.8 **Sens: 75.0**	**BAcc: 81.1** **F1-S: 78.8** Sens: 70.4

**Table 5:** Classification performance of best models using the testing set, measured by balanced accuracy (BAcc), F1-Score (F1-S), sensitivity (Sens) and specificity (Spec) (%), and corresponding confidence intervals (CI), for both independent and grouped signals.

Scenarios		Model	Independent Signals	Grouped Signals
A		Absolute FD + RAW + kNN (k = 2, distance = cityblock)	BAcc: 77.9 (95% CI = [71.1, 84.4]) F1-S: 75.2 (95% CI = [66.1, 83.1]) Sens: 67.1 (95% CI = [56.2, 78.1]) Spec: 88.6 (95% CI = [80.8, 95.6])	BAcc: 95.0 (95% CI = [83.3, 100]) F1-S: 95.2 (95% CI = [83.3, 100]) Sens: 100 (95% CI = [100, 100]) Spec: 90.0 (95% CI = [66.7, 100])
B		Imag. FD + 20 PC + kNN (k = 2, distance = cityblock)	BAcc: 62.5 (95% CI = [58.5, 66.4]) F1-S: 63.9 (95% CI = [59.2, 68.3]) Sens: 66.4 (95% CI = [60.7, 71.8]) Spec: 58.6 (95% CI = [52.8, 64.3])	BAcc: 90.0 (95% CI = [75.0, 100]) F1-S: 90.9 (95% CI = [75.0, 100]) Sens: 100 (95% CI = [100, 100]) Spec: 80.0 (95% CI = [50.0, 100])
C	H-M	TD + 19 PC + kNN (k = 1, distance = cityblock)	BAcc: 54.3 (95% CI = [47.8, 60.5]) F1-S: 65.7 (95% CI = [59.7, 71.2]) Sens: 59.7 (95% CI = [52.7, 66.5]) Spec: 48.8 (95% CI = [38.2, 59.5])	BAcc: 69.0 (95% CI = [30.0, 100]) F1-S: 76.9 (95% CI = [44.4, 100]) Sens: 71.4 (95% CI = [33.3, 100]) Spec: 66.7 (95% CI = [0, 100])
	H-M1	TD + 14 PC + SVM (kernel = RBF, γ = $2^{-1}$, C = $2^{9}$)	BAcc: 43.5 (95% CI = [36.6, 50.3]) F1-S: 52.6 (95% CI = [44.3, 60.1]) Sens: 53.6 (95% CI = [44.2, 62.6]) Spec: 33.3 (95% CI = [23.5, 43.5])	BAcc: 25.0 (95% CI = [0, 50.0]) F1-S: 44.4 (95% CI = [0, 72.7]) Sens: 50.0 (95% CI = [0, 100]) Spec: 0.0 (95% CI = [0, 0])
	H-M2	TD + 19 PC + SVM (kernel = polynomial, γ = $2^{-1}$, C = $2^{-6}$)	BAcc: 66.1 (95% CI = [58.7, 73.1]) F1-S: 57.8 (95% CI = [59.3, 75.4]) Sens: 71.4 (95% CI = [61.4, 80.9]) Spec: 60.7 (95% CI = [50.0, 71.0])	BAcc: 83.3 (95% CI = [50.0, 100]) F1-S: 85.7 (95% CI = [40.0, 100]) Sens: 100 (95% CI = [100, 100]) Spec: 66.7 (95% CI = [0, 100])

**Table 6:** Comparison with state-of-the-art classification approaches. Class type 0 vs. 1 represents a classification between no target and one target, 1 vs. 1 represents a classification between one benign and one malignant target, 0 vs. 1/2 represents a classification between no target and 1 or 2 targets.

Paper	Anatomical/Setup Representation			Classification Parameters				
	Body Part	No. of Models	View	Input Type	Class Type	Train/Validation /Test	Best Algorithm	Acc./Sens. /Spec.
[17]	Breast	-	360°	Sim. signals	0 vs. 1	2160/620/-	PCA + kNN	96.8%
[18]	Breast	15	360°	Exp. signals	0 vs. 1	43,200/4800/7200	PCA + SVM	73/67/76%
[19]	Breast	79	360°	Exp. images	0 vs. 1	1008/-/249	CNN	75/82/70%
[20]	Breast	10	360°	Exp. signals	0 vs. 1	192/96/-	PCA + LDA	85%/-/-
[25]	Breast	26	360°	Exp. signals	1 vs. 1	3456/288/-	PCA + kNN	96/92/92%
[36]	Breast	24	360°	Exp. images	1 vs. 1	21/3/-	QDA	89/77/100%
[38]	Breast	113	360°	Exp. signals	1 vs. 1	117,159/29,289/-	Adaboost	78/79/77%
[26]	Brain	200	360°	Sim. images	1 vs. 1	600/600/-	SVM	88/91/87%
[29]	Brain	300	360°	Sim. images	0 vs. 1 vs. 2	6000/60/48	CNN	98/98/99%
Ours	Axilla	80	90°	Sim. signals	1 vs. 1	1652/28/560	PCA + kNN	90/100/80%
Ours	Axilla	40	90°	Sim. signals	0 vs. 1/2	812/28/280	PCA + SVM	70/86/33%

## Data Availability

Dataset available upon request from the authors.

## Supplementary Materials

The following supporting information can be downloaded at: https://www.mdpi.com/article/10.3390/s26144466/s1, Supplementary Material S1. Parameters of ALN modelling. Table S1. Parameters for ALN modelling of all the 28 original ALN models. H means healthy, and M means metastasised. Supplementary Material S2. Dimensions of the ALN models. Table S2. Dimensions of the models 1–20 of healthy (H) ALNs and corresponding agreements with the defined morphological criteria for each type of diagnosis; Table S3. Dimensions of the models 21–40 of healthy (H) ALNs and corresponding agreements with the defined morphological criteria for each type of diagnosis; Table S4. Dimensions of the models 41–60: Models of metastasised (M) ALNs and corresponding agreements with the defined morphological criteria for each type of diagnosis; Table S5. Dimensions of the models 61–80: Models of metastasised (M) ALNs and corresponding agreements with the defined morphological criteria for each type of diagnosis.

## Abbreviations

The following abbreviations are used in this manuscript:

ALN	Axillary Lymph Node
CST	Computer Simulation Technology
CV	Cross-Validation
DT	Decision Trees
FD	Frequency Domain
FEM	Feature Extraction Method
ICH	Intracerebral Haemorrhage Stroke
IS	Ischaemic Stroke
kNN	k-Nearest Neighbours
LDA	Linear Discriminant Analysis
LOO	Leave-One-Out
ML	Machine Learning
MRI	Magnetic Resonance Imaging
MWI	Microwave Imaging
NB	Näive Bayes
PCA	Principal Component Analysis
PML	Perfectly Matched Layer
RFO	Random Forests
QDA	Quadratic Discriminant Analysis
SLNB	Sentinel Lymph Node Biopsy
SVM	Support Vector Machines
TD	Time Domain

## Appendix A. Classifier Hyperparameters

**Table A1:** Grid-search hyperparameters for each classifier.

Classifier	Hyperparameter Name	Hyperparameter Values
LDA	Amount of regularisation (γ)	10 linearly-spaced values: 0,…,1
	Linear coefficient threshold (δ)	10 logarithmically-spaced values: $10^{-2},\ldots ,10^{2}$
QDA	Amount of regularisation (γ)	10 linearly-spaced values: 0,…,1
kNN	Number of neighbours (*k*)	1,2,…,10
	Type of distance (*d*)	cityblock, chebyshev, euclidean, hamming
NB	Data distribution	normal (Gaussian), multinomial, kernel density estimate
DT	Pruning criterion	error, impurity
	Split criterion (if impurity is chosen)	Gini, twoing rule, cross entropy
RFO	Number of trees	12 linearly-spaced values: 25,…,300
	Pruning criterion	(same as DT)
	Split criterion	(same as DT)
SVM	Kernel	linear, polynomial (order 3), RBF
	kernel scale parameter (σ)	7 logarithmically-spaced values: $2^{-16},\ldots ,2^{16}$
	box constraint (*C*)	7 logarithmically-spaced values: $2^{-16},\ldots ,2^{16}$

## Appendix B. Performance Results

### Appendix B.1. Performance Results of All Tested Classifiers in the Training/Validation Set

**Table A2:** Classification performance, measured by balanced accuracy (%), for independent signals in scenario A, after classifier hyperparameter optimisation. The PCA rows correspond to the optimal results obtained using 1 to 20 principal components. N/A means the classifier was not able to converge, which can occur when there are highly correlated features or features with zero variance. The best metrics for each type of signals are highlighted in bold.

Type of Signals	FEM	LDA	QDA	kNN	NB	DT	RFO	SVM
Absolute FD	RAW	71.2	50.0	**95.7**	69.1	88.3	92.4	84.5
	F25	N/A	N/A	90.7	71.9	83.3	88.3	82.4
	PCA	74.8	73.6	95.5	74.3	85.5	91.7	86.2
Real Part FD	RAW	65.2	52.9	94.5	70.5	84.8	92.6	86.7
	F25	78.8	61.9	92.4	74.1	83.1	89.3	84.0
	PCA	72.9	75.2	**95.2**	75.2	86.9	91.4	85.2
Imaginary Part FD	RAW	64.8	46.9	**95.2**	68.8	85.0	93.1	85.2
	F25	75.0	57.1	94.0	74.0	89.1	89.5	83.8
	PCA	68.1	75.5	**95.2**	75.2	87.1	92.9	86.2
TD	RAW	62.6	55.5	92.9	74.5	85.0	87.4	84.3
	F25	N/A	N/A	92.1	70.0	81.2	87.4	84.3
	PCA	71.9	73.8	**94.5**	74.3	88.8	92.1	85.0

**Table A3:** Classification performance, measured by balanced accuracy (%), for independent signals in scenario B, after classifier hyperparameter optimisation. The PCA rows correspond to the optimal results obtained using 1 to 20 principal components. N/A means the classifier was not able to converge, which can occur when there are highly correlated features or features with zero variance. The best metrics for each type of signals are highlighted in bold.

Type of Signals	FEM	LDA	QDA	kNN	NB	DT	RFO	SVM
Absolute FD	RAW	58.3	50.2	95.5	50.2	82.9	91.5	91.0
	F25	N/A	N/A	88.5	45.3	81.0	87.9	81.0
	PCA	56.3	57.2	**96.0**	61.4	85.4	92.7	91.6
Real Part FD	RAW	61.8	49.6	95.3	49.6	82.6	91.1	90.0
	F25	44.6	44.6	82.6	49.9	83.2	88.7	77.3
	PCA	61.1	56.8	**96.4**	63.8	83.8	91.8	92.5
Imag. Part FD	RAW	60.4	50.0	95.5	50.0	83.7	92.3	90.5
	F25	49.6	50.1	93.0	50.0	84.3	89.3	86.3
	PCA	61.5	59.2	**96.7**	60.2	85.7	93.0	95.0
TD	RAW	55.6	50.5	95.0	50.5	85.7	87.5	90.5
	F25	N/A	N/A	90.3	51.8	82.7	86.8	84.9
	PCA	45.2	60.7	**95.6**	64.7	85.1	92.1	91.9

**Table A4:** Classification performance, measured by balanced accuracy (%), for independent signals in scenario C, after classifier hyperparameter optimisation when using PCA-X as FEM. The best metrics for each type of signals are highlighted in bold.

Comparison	Type of Signals	LDA	QDA	kNN	NB	DT	RFO	SVM
H-M	Absolute FD	55.6	66.7	74.6	66.8	69.4	79.3	**81.8**
	Real Part FD	66.7	66.7	72.1	66.7	68.3	77.1	**79.2**
	Imag. Part FD	68.2	66.7	72.1	66.7	67.6	75.9	**78.7**
	TD	66.7	66.7	78.1	66.7	71.1	81.3	**84.0**
H-M1	Absolute FD	51.6	58.6	69.8	58.9	63.8	69.3	**73.0**
	Real Part FD	56.6	58.9	65.0	56.3	61.3	68.0	**70.2**
	Imag. Part FD	50.4	57.1	65.0	58.8	62.0	68.4	**72.0**
	TD	34.1	56.4	69.8	59.5	63.9	70.9	**75.5**
H-M2	Absolute FD	52.7	57.3	**78.4**	55.0	66.6	77.0	76.4
	Real Part FD	49.5	54.1	74.8	52.7	66.4	72.7	**75.5**
	Imag. Part FD	50.7	55.2	75.9	54.1	68.2	75.5	**76.8**
	TD	31.1	55.4	78.2	58.2	69.1	76.1	**81.1**

### Appendix B.2. Performance Results When Considering Other Methodology for Scenario B

**Table A5:** Classification performance, measured by balanced accuracy (%), for both independent and grouped signals in scenario B, after classifier hyperparameter optimisation and when considering three planes in training and the remaining one in validation set. The absolute part of FD signals were the used type of signals. The PCA rows correspond to the optimal results obtained using 1 to 20 principal components.

Type of Classification	FEM	All Planes		Plane in Separate	
		kNN	DT	kNN	DT
Independent signals	RAW	95.5	82.9	71.6	52.6
	F25	88.5	81.0	63.4	50.7
	PCA	96.0	85.4	78.3	55.5
Grouped signals	RAW	98.3	95.0	100.0	61.7
	F25	98.3	96.7	91.7	50.0
	PCA	98.3	96.7	100.0	73.3

**Table A6:** Classification performance, measured by balanced accuracy (%), for both independent and grouped signals in scenario A, after classifier hyperparameter optimisation and when considering separating augmented ALNs in different training and validation sets. The absolute part of FD signals were the used type of signals. The PCA rows correspond to the optimal results obtained using 1 to 20 principal components.

Type of Classification	FEM	No Separation		Separation	
		kNN	DT	kNN	DT
Independent signals	RAW	95.7	88.3	91.7	87.1
	F25	90.7	83.3	84.3	81.0
	PCA	95.5	85.5	92.9	85.0
Grouped signals	RAW	98.3	96.7	96.7	95.0
	F25	95.0	93.3	91.7	93.3
	PCA	98.3	91.7	98.3	90.0

## References

[1] The Global Cancer Observatory—World Health Organization Breast Cancer Fact Sheets. http://gco.iarc.fr/today.

[2] Siegel R.L., Miller K.D., Wagle N.S., Jemal A. (2023). Cancer statistics, 2023. CA Cancer J. Clin..

[3] DeSantis C.E., Ma J., Gaudet M.M., Newman L.A., Miller K.D., Sauer A.G., Jemal A., Siegel R.L. (2019). Breast Cancer Statistics, 2019. CA Cancer J. Clin..

[4] Rahbar H., Partridge S.C., Javid S.H., Lehman C.D. (2012). Imaging Axillary Lymph Nodes in Patients with Newly Diagnosed Breast Cancer. Curr. Probl. Diagn. Radiol..

[5] Valente S.A., Levine G.M., Silverstein M.J., Rayhanabad J.A., Weng-Grumley J.G., Ji L., Holmes D.R., Sposto R., Sener S.F. (2012). Accuracy of predicting axillary lymph node positivity by physical examination, mammography, ultrasonography, and magnetic resonance imaging. Ann. Surg. Oncol..

[6] Aktaş A., Gürleyik M.G., Aydın Aksu S., Aker F., Güngör S. (2022). Diagnostic Value of Axillary Ultrasound, MRI, and 18F-FDG-PET/CT in Determining Axillary Lymph Node Status in Breast Cancer Patients. Eur. J. Breast Health.

[7] Mansel R.E., Fallow L., Kissin M., Goyal A., Newcombe R.G., Dixon J.M., Yiangou C., Horgan K., Bundred N., Monypenny I. (2006). Randomized Multicenter Trial of Sentinel Node Biopsy Versus Standard Axillary Treatment in Operable Breast Cancer: The ALMANAC Trial. J. Natl. Cancer Inst..

[8] Kim G.R., Choi J.S., Han B.K., Lee J.E., Nam S.J., Ko E.Y., Ko E.S., Lee S.K. (2018). Preoperative axillary US in early-stage breast cancer: Potential to prevent unnecessary axillary lymph node dissection. Radiology.

[9] Viale G., Zurrida S., Maiorano E., Mazzarol G., Pruneri G., Paganelli G., Maisonneuve P., Veronesi U. (2005). Predicting the status of axillary sentinel lymph nodes in 4351 patients with invasive breast carcinoma treated in a single institution. Cancer.

[10] Shere M., Lyburn I., Sidebottom R., Massey H., Gillett C., Jones L. (2019). MARIA^®^ M5: A Multicentre Clinical Study to Evaluate the Ability of the Micrima Radio-Wave Radar Breast Imaging System (MARIA^®^) to Detect Lesions in the Symptomatic Breast. Eur. J. Radiol..

[11] Vasquez J.A., Scapaticci R., Turvani G., Bellizzi G., Rodriguez-Duarte D.O., Joachimowicz N., Duchêne B., Tedeschi E., Casu M.R., Crocco L. (2020). A Prototype Microwave System for 3D Brain Stroke Imaging. Sensors.

[12] Godinho D.M., Felício J.M., Fernandes C.A., Conceição R.C. (2022). Experimental Evaluation of an Axillary Microwave Imaging System to Aid Breast Cancer Staging. IEEE J. Electromagn. RF Microw. Med. Biol..

[13] Savazzi M., Abedi S., Ištuk N., Joachimowicz N., Roussel H., Porter E., O’ Halloran M., Costa J.R., Fernandes C.A., Felício J.M. (2020). Development of an Anthropomorphic Phantom of the Axillary Region for Microwave Imaging Assessment. Sensors.

[14] Choi J.W., Cho J., Lee Y., Yim J., Kang B., Ki K.O., Woo H.J., Hee J.K., Cheon C., Lee H.D. (2004). Microwave Detection of Metastasized Breast Cancer Cells in the Lymph Node; Potential Application for Sentinel Lymphadenectomy. Breast Cancer Res. Treat..

[15] Cameron T.R., Okoniewski M., Fear E.C. A Preliminary Study of the Electrical Properties of Healthy and Diseased Lymph Nodes. Proceedings of the 14th International Symposium on Antenna Technology and Applied Electromagnetics & the American Electromagnetics Conference (ANTEM/AMEREM).

[16] Godinho D.M., Felício J.M., Castela T., Silva N.A., Orvalho M.D.L., Fernandes C.A., Conceição R.C. (2021). Development of MRI-Based Axillary Numerical Models and Estimation of Axillary Lymph Node Dielectric Properties for Microwave Imaging. Med. Phys..

[17] Byrne D., O’Halloran M., Glavin M., Jones E. (2011). Breast cancer detection based on differential ultrawideband microwave radar. Prog. Electromagn. Res. M.

[18] Santorelli A., Porter E., Kirshin E., Liu Y.J., Popovic M. (2014). Investigation of Classifiers for Tumor Detection with an Experimental Time-Domain Breast Screening System. Prog. Electromagn. Res..

[19] Reimer T., Pistorius S. (2021). The Diagnostic Performance of Machine Learning in Breast Microwave Sensing on an Experimental Dataset. IEEE J. Electromagn. RF Microw. Med. Biol..

[20] Martins R.A., Felicio J.M., Costa J.R., Fernandes C.A. Comparison of Slot-based and Vivaldi Antennas for Breast Tumor Detection using Machine Learning and Microwave Imaging Algorithms. Proceedings of the 15th European Conference on Antennas and Propagation (EuCAP).

[21] Davis S.K., Van Veen B.D., Hagness S.C., Kelcz F. (2008). Breast Tumor Characterization Based on Ultrawideband Microwave Backscatter. IEEE Trans. Biomed. Eng..

[22] Conceição R.C., O’Halloran M., Glavin M., Jones E. (2010). Support Vector Machines for the Classification of Early-Stage Breast Cancer Based on Radar Target Signatures. Prog. Electromagn. Res. B.

[23] McGinley B., O’Halloran M., Conceicao R.C., Morgan F., Glavin M., Jones E. (2010). Spiking Neural Networks for Breast Cancer Classification Using Radar Target Signatures. Prog. Electromagn. Res. C.

[24] Oliveira B.L., Godinho D., O’Halloran M., Glavin M., Jones E., Conceição R.C. (2018). Diagnosing Breast Cancer with Microwave Technology: Remaining Challenges and Potential Solutions with Machine Learning. Diagnostics.

[25] Conceição R.C., Medeiros H., Godinho D.M., O’Halloran M., Rodriguez-Herrera D., Flores-Tapia D., Pistorius S. (2020). Classification of Breast Tumor Models with a Prototype Microwave Imaging System. Med. Phys..

[26] Guo L., Abbosh A. (2018). Stroke localization and classification using microwave tomography with k-means clustering and support vector machine. Bioelectromagnetics.

[27] Zhu G., Bialkowski A., Guo L., Mohammed B., Abbosh A. (2021). Stroke Classification in Simulated Electromagnetic Imaging Using Graph Approaches. IEEE J. Electromagn. RF Microw. Med. Biol..

[28] Pokorny T., Vrba J., Fiser O., Vrba D., Drizdal T., Novak M., Tosi L., Polo A., Salucci M. (2023). On the Role of Training Data for SVM-Based Microwave Brain Stroke Detection and Classification. Sensors.

[29] Hossain A., Islam M.T., Rahman T., Chowdhury M.E., Tahir A., Kiranyaz S., Mat K., Beng G.K., Soliman M.S. (2023). Brain Tumor Segmentation and Classification from Sensor-Based Portable Microwave Brain Imaging System Using Lightweight Deep Learning Models. Biosensors.

[30] Saied I.M., Arslan T., Chandran S. (2022). Classification of Alzheimer’s Disease Using RF Signals and Machine Learning. IEEE J. Electromagn. RF Microw. Med. Biol..

[31] Ullah R., Dong Y., Arslan T., Chandran S. (2023). A Machine Learning-Based Classification Method for Monitoring Alzheimer’s Disease Using Electromagnetic Radar Data. IEEE Trans. Microw. Theory Tech..

[32] Teo J., Chen Y., Soh C.B., Gunawan E., Low K.S., Putti T.C., Wang S.C. (2010). Breast lesion classification using ultrawideband early time breast lesion response. IEEE Trans. Antennas Propag..

[33] Chen Y., Craddock I.J., Kosmas P. (2010). Feasibility study of lesion classification via contrast-agent-aided UWB breast imaging. IEEE Trans. Biomed. Eng..

[34] Standring S. (2016). Blood, Lymphoid Tissues and Haemopoiesis. Gray’s Anatomy: The Anatomical Basis of Clinical Practice.

[35] Chen C.F., Zhang Y.L., Cai Z.L., Sun S.M., Lu X.F., Lin H.Y., Liang W.Q., Yuan M.H., Zeng D. (2019). Predictive Value of Preoperative Multidetector-Row Computed Tomography for Axillary Lymph Nodes Metastasis in Patients with Breast Cancer. Front. Oncol..

[36] Moloney B.M., Mcanena P.F., Elwahab S.M., Fasoula A., Duchesne L., Cano J.D., Glynn C., O’connell A., Ennis R., Lowery A.J. (2021). The Wavelia Microwave Breast Imaging system-tumour discriminating features and their clinical usefulness. Br. J. Radiol..

[37] Fasoula A., Duchesne L., Cano J.D.G., Moloney B.M., Elwahab S.M., Kerin M.J. (2021). Automated breast lesion detection and characterization with the wavelia microwave breast imaging system: Methodological proof-of-concept on first-in-human patient data. Appl. Sci..

[38] Janjic A., Akduman I., Cayoren M., Bugdayci O., Aribal M.E. (2022). Microwave Breast Lesion Classification—Results from Clinical Investigation of the SAFE Microwave Breast Cancer System. Acad. Radiol..

[39] Elnaggar A.H., El-Hameed A.S.A., Yakout M.A., Areed N.F.F. (2024). Machine Learning for Breast Cancer Detection with Dual-Port Textile UWB MIMO Bra-Tenna System. Information.

[40] Taha B., Liza F.R., Masud M.A., Bepery C., Islam M.T., Samsuzzaman M. BrainVisionNet: A Deep Learning-based Approach to Evaluate the Potential of Microwave Imaging for Classification of Brain Tumors. Proceedings of the 2023 International Conference on Next-Generation Computing, IoT and Machine Learning (NCIM).

[41] Shao W. (2025). Machine Learning in Microwave Medical Imaging and Lesion Detection. Diagnostics.

[42] Silva T.M.M., Conceição R.C., Godinho D.M. (2025). Machine and deep learning applied to medical microwave imaging: A scoping review from reconstruction to classification. Prog. Biomed. Eng..

[43] Godinho D.M. (2022). Microwave Imaging to Improve Breast Cancer Diagnosis. Ph.D. Dissertation.

[44] Baltzer P.A., Dietzel M., Burmeister H.P., Zoubi R., Gajda M., Camara O., Kaiser W.A. (2011). Application of MR mammography beyond local staging: Is there a potential to accurately assess axillary lymph nodes? Evaluation of an extended protocol in an initial prospective study. AJR Am. J. Roentgenol..

[45] Dudea S.M., Lenghel M., Botar-Jid C., Vasilescu D., Duma M. (2012). Ultrasonography of superficial lymph nodes: Benign vs. malignant. Med. Ultrason..

[46] Mao Y., Hedgire S., Harisinghani M. (2014). Radiologic Assessment of Lymph Nodes in Oncologic Patients. Curr. Radiol. Rep..

[47] Felício J.M., Bioucas-Dias J.M., Costa J.R., Fernandes C.A. (2019). Antenna Design and Near-Field Characterization for Medical Microwave Imaging Applications. IEEE Trans. Antennas Propag..

[48] Bentel G.C., Marks L.B., Hardenbergh P.H., Prosnitz L.R. (2000). Variability of the depth of supraclavicular and axillary lymph nodes in patients with breast cancer: Is a posterior axillary boost field necessary?. Int. J. Radiat. Oncol. Biol. Phys..

[49] Costa J.R., Medeiros C.R., Fernandes C.A. (2009). Performance of a Crossed Exponentially Tapered Slot Antenna for UWB Systems. IEEE Trans. Antennas Propag..

[50] Dassault Systemes CST—Computer Simulation Technology. https://www.3ds.com/products-services/simulia/products/cst-studio-suite/.

[51] Conceição R.C., Godinho D.M. Extracting Features from Multistatic Signals in a Radar Microwave Imaging System for Breast Cancer Detection. Proceedings of the 2nd URSI AT-RASC.

[52] Godinho D.M., Silva C., Baleia C., Felício J.M., Castela T., Silva N.A., Orvalho M.L., Fernandes C.A., Conceição R.C. (2022). Modelling level I Axillary Lymph Nodes depth for Microwave Imaging. Phys. Medica.

[53] Somner J.E.A., Dixon J.M.J., Thomas J.S.J. (2004). Node retrieval in axillary lymph node dissections: Recommendations for minimum numbers to be confident about node negative status. J. Clin. Pathol..

[54] Rosen P.P., Lesser M.L., Kinne D.W., Beattie E.J. (1983). Discontinuous or ’skip’ metastases in breast carcinoma: Analysis of 1228 axillary dissections. Ann. Surg..

