# Supplementary material for: Machine Learning Classification of Axillary Lymph Nodes Using Microwave Signals
Source: Sensors (Basel). 2026 Jul 14;26(14):4466. doi: 10.3390/s26144466 (PMC13431324; doi:10.3390/s26144466)
Supplement: Supplementary file 1 [file sensors-26-04466-s001.zip › sensors-4387428-supplementary.pdf]

Supplementary Materials: Machine Learning Classification of Axillary Lymph Nodes using Microwave Signals

Daniela M. Godinho[1,*], João M. Felício[2], Carlos A. Fernandes[2] and Raquel C. Conceição[1]

Supplementary Material 1. Parameters of ALN modelling

Table S1. Parameters for ALN modelling of all the 28 original ALN models. H means Healthy and M means Metastasised.

ALN model	Equations parameters					Rescale
	a	b	c	d	θ_0	
H1	$\pi/3.8$	0.4	0.9	1.3	$1.9\pi/4$	-
H2	$\pi/5$	0.4	0.9	1.3	$1.9\pi/4$	x=0.8; y=0.9
H3	$\pi/4$	0.4	0.8	1.9	$1.95\pi/4$	-
H4	$\pi/4$	0.4	0.9	1.3	$1.9\pi/4$	x=0.5; y=0.7
H5	$\pi/2$	0.9	0.9	3	$2\pi/$	-
H6	$\pi/2$	0.9	0.5	1	$\pi/2$	-
H7	$\pi/5$	1	0.3	2	$2\pi/5$	-
H8	$\pi/2$	0.6	0.7	0.5	$\pi/10$	-
H9	$3\pi/10$	0.5	0.7	1	$\pi/2$	-
H10	$\pi/6$	0.6	0.9	1.3	$1.9\pi/4$	-
H11	$\pi/6$	0.5	0.7	1	$2\pi/5$	-
H12	$2\pi/3$	0.6	0.8	0.8	$\pi/2$	-
H13	$\pi/6$	0.7	0.5	0.8	$\pi/2$	-
H14	$\pi/6$	0.6	0.2	0.8	$\pi/2$	-
M1	$\pi/7$	0.3	0.4	2.5	$1.8\pi/4$	-
M2	$1.9\pi/4$	0.3	0.5	1.3	$1.9\pi/4$	x=3; y=3; z=1.8
M3	$\pi/5$	0.4	0.3	1.5	$1\pi/2$	x=3; y=3; z=2.4
M4	$\pi/5$	0.4	0.3	1.5	$1.5\pi/4$	x=1.5; y=1.5; z=1.5
M5	$\pi/2$	0.3	1	3	$2\pi/$	-
M6	$\pi/2$	0.01	0.01	0.01	2π	-
M7	$\pi/5$	0	0.5	0.5	$2\pi/3$	-
M8	$\pi/6$	0	0.5	0.2	$2\pi/3.5$	-
M9	$\pi/2$	0.3	1	1	$2\pi/$	-
M10	$\pi/2$	0.6	0.3	1	$2\pi/$	-
M11	$\pi/2$	0.2	0.8	0.8	2π	-
M12	$\pi/2$	0.2	0.8	0.8	$2\pi/2.5$	-
M13	$\pi/2$	0.2	0.5	0.5	$\pi/2$	-
M14	$\pi/6$	0.6	0.2	0.8	$\pi/2$	x=0.9; z=0.6

Supplementary Material 2. Dimensions of the ALN models

Table S2. Dimensions of the models 1-20 of Healthy (H) ALNs and corresponding agreements with the defined morphological criteria for each type of diagnosis.

ALN	Dimensions		Criteria		Original
	L	S	$L/S \geq 7$	$S \leq 9$	Model
#1	10	5	TRUE	TRUE	H1
#2	10	5	TRUE	TRUE	H1 rotated
#3	10	5	TRUE	TRUE	H2
#4	9	4	TRUE	TRUE	H2 rotated and resized
#5	11	4	TRUE	TRUE	H3 resized
#6	11	6	TRUE	TRUE	H3 rotated and resized
#7	10	3	TRUE	TRUE	H4
#8	16	3	TRUE	TRUE	H4 rotated and resized
#9	20	9	TRUE	TRUE	H1 rotated and resized
#10	20	5	TRUE	TRUE	H2 rotated and resized
#11	20	8	TRUE	TRUE	H3 rotated and resized
#12	20	6	TRUE	TRUE	H4 rotated and resized
#13	11	4	TRUE	TRUE	H5 resized
#14	16	6	TRUE	TRUE	H5 resized and rotated
#15	18	6	TRUE	TRUE	H5 resized and rotated
#16	10	3	TRUE	TRUE	H6
#17	15	3	TRUE	TRUE	H6 resized and rotated
#18	20	3	TRUE	TRUE	H6 resized and rotated
#19	13	4	TRUE	TRUE	H7
#20	13	5	TRUE	TRUE	H7 resized and rotated

https://doi.org/10.3390/s1010000

Table S3. Dimensions of the models 21-40 of Healthy (H) ALNs and corresponding agreements with the defined morphological criteria for each type of diagnosis.

ALN	Dimensions		Criteria		Original
	L	S	$L/S \geq 7$	$S \leq 9$	Model
#21	20	5	TRUE	TRUE	H7 resized and rotated
#22	13	3	TRUE	TRUE	H8 resized
#23	14	3	TRUE	TRUE	H8 resized and rotated
#24	20	6	TRUE	TRUE	H8 resized and rotated
#25	10	5	TRUE	TRUE	H9
#26	15	6	TRUE	TRUE	H9 resized and rotated
#27	19	6	TRUE	TRUE	H9 resized and rotated
#28	10	4	TRUE	TRUE	H10
#29	14	6	TRUE	TRUE	H10 resized and rotated
#30	19	7	TRUE	TRUE	H10 resized and rotated
#31	10	5	TRUE	TRUE	H11
#32	5	4	FALSE	TRUE	H11 resized and rotated
#33	26	10	TRUE	FALSE	H11 resized and rotated
#34	10	3	TRUE	TRUE	H12 rotated
#35	15	4	TRUE	TRUE	H12 resized and rotated
#36	20	6	TRUE	TRUE	H12 resized and rotated
#37	10	4	TRUE	TRUE	H13 rotated
#38	12	7	TRUE	TRUE	H13 resized and rotated
#39	8	7	FALSE	TRUE	H13 resized and rotated
#40	10	6	FALSE	TRUE	H14

https://doi.org/10.3390/s1010000

Table S4. Dimensions of the models 41-60 models of Metastasised (M) ALNs and corresponding agreements with the defined morphological criteria for each type of diagnosis.

**ALN**	**Dimensions**		**Criteria**		**Original**
	L	S	$L/S \geq 7$	$S \leq 9$	**Model**
#41	13	10	FALSE	FALSE	M1
#42	13	10	FALSE	FALSE	M1 rotated
#43	19	18	FALSE	FALSE	M2
#44	12	11	FALSE	FALSE	M2 rotated and resized
#45	17	13	FALSE	FALSE	M3 resized
#46	17	13	FALSE	FALSE	M3 rotated and resized
#47	15	11	FALSE	FALSE	M4
#48	15	11	FALSE	FALSE	M4 rotated
#49	20	15	FALSE	FALSE	M1 rotated and resized
#50	20	18	FALSE	FALSE	M2 rotated and resized
#51	18	13	FALSE	FALSE	M3 rotated and resized
#52	19	14	FALSE	FALSE	M4 rotated and resized
#53	12	10	FALSE	FALSE	M5 resized
#54	11	10	FALSE	FALSE	M5 rotated and resized
#55	17	11	FALSE	FALSE	M5 rotated and resized
#56	10	10	FALSE	FALSE	M6
#57	14	12	FALSE	FALSE	M6 resized
#58	18	13	FALSE	FALSE	M6 resized
#59	11	10	FALSE	FALSE	M7 resized
#60	14	10	FALSE	FALSE	M7 resized and rotated

Table S5. Dimensions of the models 61-80 models of Metastasised (M) ALNs and corresponding agreements with the defined morphological criteria for each type of diagnosis.

**ALN**	**Dimensions**		**Criteria**		**Original**
	L	S	$L/S \geq 7$	$S \leq 9$	**Model**
#61	19	14	FALSE	FALSE	M7 resized and rotated
#62	11	11	FALSE	FALSE	M8 resized
#63	13	11	FALSE	FALSE	M8 resized and rotated
#64	17	12	FALSE	FALSE	M8 resized and rotated
#65	10	10	FALSE	FALSE	M9 resized
#66	13	10	FALSE	FALSE	M9 resized and rotated
#67	19	13	FALSE	FALSE	M9 resized and rotated
#68	11	10	FALSE	FALSE	M10 resized
#69	15	10	FALSE	FALSE	M10 resized and rotated
#70	17	11	FALSE	FALSE	M10 resized and rotated
#71	12	10	FALSE	FALSE	M11 resized
#72	15	10	FALSE	FALSE	M11 resized and rotated
#73	17	13	FALSE	FALSE	M11 resized and rotated
#74	11	10	FALSE	FALSE	M12 resized and rotated
#75	15	12	FALSE	FALSE	M12 resized and rotated
#76	20	14	FALSE	FALSE	M12 resized
#77	13	10	FALSE	FALSE	M13 resized and rotated
#78	13	13	FALSE	FALSE	M13 resized and rotated
#79	16	13	FALSE	FALSE	M13 resized and rotated
#80	11	10	FALSE	FALSE	H14 resized and rotated

3

https://doi.org/10.3390/s1010000
